# Tumour modelling using viral vectors

**DOI:** 10.18632/oncotarget.4387

**Published:** 2015-06-08

**Authors:** Ian J. Frew

**Affiliations:** Institute of Physiology and Zurich Center for Integrative Human Physiology, University of Zurich, Zurich, Switzerland

Human tumours typically harbour multiple mutations in tumour suppressor genes and oncogenes. Histologically identical tumours in different patients often exhibit different combinations of genetic alterations and it is now also evident that considerable genetic heterogeneity can exist between cells within individual tumours. A major ongoing research challenge remains to determine the functional significance of this enormous genetic diversity and heterogeneity in terms of impact on tumour phenotypes and responses to therapies. Autochthonous mouse models of human tumours have provided many insights into the combinations of genetic alterations that are causal to tumour initiation and progression in different tissues. However, conventional germline-based genetic approaches, such as the generation of compound mutant mice based on transgenics and knockouts, are limited by the time and cost associated with extensive intercrossing of mouse lines. Faster and more powerful genetic tools are required to accelerate the functional annotation of cancer mutational cataloguing studies. Excitingly, several new viral vector-mediated genetic approaches offer the ability to directly modify the genome of somatic cells in mouse tissues and these have recently been applied to the rapid generation of complex mouse tumour models that harbour multiple genetic changes.

The use of lentiviral gene delivery vectors for cancer modelling studies offers the advantage that the proviral DNA integrates into the genome of infected cells, providing heritability of the introduced genetic alterations. Injections of lentiviruses that co-express shRNAs against *Trp53* and *Nf1* or that co-express oncogenic *Hras* and shRNA against *Trp53* were employed to generate autochthonous mouse models of glioma [1]. This study provided proof-of-principle that the lentiviral approach is a feasible tool for cancer modelling. Building upon this idea, we recently generated the MuLE lentiviral system (http://www.addgene.org/kits/mule-system/) that provides a highly flexible genetic toolbox allowing combinatorial gene knockdown, knockout, mutation or overexpression, together with tagging of infected cells with useful markers such as luciferase or fluorescent proteins [2]. We demonstrated the utility of this system for generating genetically complex autochthonous tumour models by showing that intramuscular injection of ecotropic MuLE lentiviruses expressing luciferase, oncogenic *Hras* and shRNAs against *Cdkn2a* or *Trp53* or against both *Trp53* and *Pten*, induced the formation of pleomorphic sarcomas that could be quantitatively monitored using luciferase-based imaging [2]. The histology of these tumours was indistinguishable from the pleomorphic sarcomas that arose through transgenic or adenoviral Cre-mediated genetic deletion of *Trp53* and activation of oncogenic *Kras* in germline-modified mice [3,4], validating that the MuLE-mediated somatic genetics approach can rapidly and faithfully recapitulate tumour models obtained using conventional genetic strategies.

Several genetically complex lung cancer models in mice have recently been generated using different types of viral vectors. Adenovirus and Adeno-associated virus (AAV) DNA remains episomal in infected mouse cells and is lost by dilution upon repeated cell division, which has historically limited the use of these vectors in tumour modelling. However, in the context of the CRISPR/Cas9 system, the episomal nature of viral infection is in fact advantageous as the introduced viral DNA represents a template that can be used for homology directed repair following Cas9-induced DNA double strand breakage. Taking advantage of transgenic mice expressing Cre-activatable Cas9, the intratracheal delivery of AAV9 viruses expressing Cre, luciferase, sgRNAs against *Trp53*, *Lkb1* and *Kras*, together with a template for homology directed repair to insert an oncogenic *Kras* mutant into the endogenous locus, allowed the generation of lung tumours driven by three different genetic mutations [5]. In another model, intratracheal delivery of lentiviruses expressing Cre, Cas9 and sgRNAs against different tumour suppressor genes into mice with conditional *Trp53* and oncogenic *Kras* alleles provided a series of lung cancer models with complex genetic backgrounds [6]. Finally, infection of lung epithelia using an adenoviral vector expressing two sgRNAs and Cas9 allowed the generation of a lung cancer model driven by the *Eml4-Alk* chromosomal rearrangement [7].

These studies collectively highlight the new genetic power that is offered by the use of viral vector-mediated somatic genetics in wild type or germline modified mice. By employing different types of lentiviral, retroviral, adenoviral and AAV vectors, together with the use of cell-type specific promoters or infection of germline-modified mice that allow cell type-specific Cre, tTA or rtTA expression, it should be possible to specifically direct genetic modulations to diverse somatic cell types. The ability to clone libraries (eg. shRNA or sgRNA) into these viral expression vectors will pave the way for *in vivo* screens based on combinatorial genetics and will potentially allow experiments that better mimic intratumoural genetic heterogeneity. These new genetic tools will assist researchers in tackling the challenges of understanding the functional implications of the genetic complexities of human malignancies.
